# Dry Dressing for the Umbilical Cord

**Published:** 1885-03

**Authors:** J. G. Hopkins

**Affiliations:** Thomasville, Ga.


### DRY DRESSING FOR UMBILICAL CORD.

As I have witnessed no little suffering on the part of infants, resulting from carelessness in the management of the cord dressed in the usual way, I was induced to adopt another method, which has proven a great success. It does away with filthy grease, dried and stiff cloths, which stick to and excoriate the skin, is far neater, cleaner, and in every way more satisfactory. I simply dry thoroughly the stump, after the child has been bathed and is ready for its clothes, wrap it in a strip of cloth about a foot long, a little wider than the length of the cord, dry well the abdomen and powder it with starch, arrow-root or some such substance, and apply the band in the usual way.

After the first few turns of the strip around the cord, it is folded upon itself several times, thus forming something of a pad to interpose between the cord and abdomen.

Since first using this, I have never resorted to the old practice of grease, scorched linen, etc. Try it, and your experience will but verify my assertions.

Respectfully,

J. G. HOPKINS.

*Thomasville, Ga., February, 1885.*

